# Perspectives of Indian speech language pathologists towards assessing adolescents with language impairments

**DOI:** 10.1590/2317-1782/20232022005

**Published:** 2023-08-07

**Authors:** Rohana Muralidharan Nair, Sudhin Karuppali

**Affiliations:** 1 Department of Audiology and Speech Language Pathology, Kasturba Medical College, Mangalore, Manipal Academy of Higher Education, Light house hill road, Mangalore, Karnataka, India - 575001

**Keywords:** Adolescent, Assessment, Perspectives, Language, Development

## Abstract

**Purpose:**

Perspective-based studies have been carried out on health professionals to create clinical implications that will positively impact the healthcare system. There are no such studies exploring the perspectives of Indian speech language pathologists (SLPs) towards handling adolescents with language impairments. Therefore, the current study aims to explore the perspectives of Indian SLPs on the assessment of adolescent language.

**Methods:**

The study followed a cross-sectional study design following a non-random convenient sampling procedure. A total number of 102 SLPs participated in the study. Phase 1 comprised developing a questionnaire to identify the perspectives of SLPs towards the assessment of adolescents with language impairments. A total of 9 questions were formulated for the same. Phase 2 included the data collection which was conducted through an online survey. Phase 3 focused on the data analysis. Descriptive statistics were to determine the mean and SD for continuous variables, and frequency and percentage for discrete variables.

**Results:**

The current study results indicated significant disparities in the perspectives of SLPs towards adolescent language assessment. An overall level of poor awareness and a superficial understanding of the core area (about adolescence, and the areas and tools for assessment) was evident.

**Conclusion:**

Understanding the perspectives of SLPs towards adolescent language assessment is critical in paving the way for future clinical development and research.

## INTRODUCTION

The precise onset and offset of adolescence have always been challenging to determine ^(1)^. This period is primarily manifested by developments in biological, linguistic, socio-behavioral, novelty-seeking, and risk-taking behaviors. The timings of these transitions vary with the nutritional status, sociocultural values, and economic circumstances of the individuals^(2)^. Although language development during childhood is quite explicit, the same may not be true in adolescence, wherein linguistic growth is subtle and unnoticeable^(3)^. Adolescents with persisting language impairments are often overlooked, implying an immense need to assess these individuals^(4)^. Utley and Hopf^(5)^ estimate a high prevalence (between 16% and 92%) of language impairments in adolescents, with the numbers not only revealing the linguistic domain per se, but a more comprehensive range of other communicative aspects as well. A general lack of awareness has been reported among the general population and working professionals (social workers, teachers, and other professionals who work with adolescents) about the existence of language impairments in adolescents^(6)^. This has resulted in a steep decline in the follow-ups of such individuals, leaving them with an unattended and unnoticeable language disability^(4)^. The assessment of adolescent language can be performed using certain standardized western clinical tools, with only one recent tool^(4)^ available for the Indian population. Other assessment methods include the use of tasks that focus on expository discourse^(7,8)^ both in written^(7,8)^ and spoken modalities^(9)^, conversation^(9)^, morphological derivations^(10)^, syntactic structures^(7)^, and narratives^(11)^.

Speech Language Pathologists (SLPs) provide services in diverse settings, with a high probability of encountering communicatively challenged individuals from various age groups. It, therefore, becomes highly imperative that SLPs be well equipped with the know-how to handle adolescents whose language structures that are quite complex to be determined. Nippold^(12)^ recommends an increased collaboration between SLPs and other professionals in schools, and to instil changes to training programs to better prepare future SLPs to address adolescents with communication disabilities. This has called upon the estimation of the level of preparedness of SLPs towards service delivery for adolescents. Such perspective-based surveys have been carried out among health professionals to get an insight into their outlook on a particular subject, thereby creating clinical implications that positively impact the healthcare system. Ehren^(13)^ studied the contribution of SLPs toward the academic success of high school students in Kansas City, Missouri, US. The author expressed the effectiveness of speech and language intervention, and how it contributed to the success of adolescents, without which they were unable to succeed in either oral and/or written communication. He concluded by indicating that SLPs needed to extend their scope of practice toward this population, as their success in academics and life solely depended on the outcome of their school years. Hollands et al.^(14)^ surveyed Australian SLPs predominantly working with adolescents (aged between 12 and 18 years) with oral language difficulties, and found that in spite of existence of policies on service delivery for adolescents, there still did exist restrictions based on their age. According to them, 48% of SLPs found adolescent language intervention to be the responsibility of school-based SLPs, while 19% believed it to be a shared responsibility between school and health-based SLPs. Surprisingly, a small group of SLPs claimed adolescents were not required to go to health-based set-ups for language intervention as it was deemed inappropriate. With this current understanding of the perspectives of SLPs towards the service delivery for adolescents with language impairments, it becomes extremely vital to explore the same in the Indian context, considering the cultural and linguistic diversity of the country.

The field of audiology and speech language pathology is relatively new in India compared to the western countries and is regulated by the Rehabilitation Council of India (RCI). The RCI currently regulates six rehabilitation programs in the field of speech and hearing - Bachelors in Audiology and Speech Language Pathology (BASLP), M.Sc in Speech Language Pathology [M.Sc (SLP)], M.Sc in Audiology [M.Sc (Audiology)], Diploma in Hearing Language and Speech (DHLS), Diploma in Hearing Aid Repair and Ear Mould Technology, and Post Graduate Diploma in Auditory Verbal Therapy. A total of 49 RCI accredited institutes/organizations provide academic programs on speech and hearing throughout India. As per RCI, a professional must possess a minimum education in either BASLP or DHLS to practice as a speech language pathologist or an audiologist in India. Although certain work environments prefer to have professionals dedicated to either solely providing audiology services or SLP services, a large proportion of Indian employers prefer to have equally competent (in both disciplines) professionals. Hence estimating the proportion of audiologists versus SLPs in India is highly challenging. However, as of February 2022, there exists 2866 registered audiologists and SLPs in India. This figure may be higher, considering the proportion of fresh graduates in speech and hearing entering the health sector on an annual basis. The SLP professionals generally work in private clinics, academic institutions with clinical services, rehabilitation centers, mental health facilities, regular or special schools, and private/government hospitals. Considering the diversity of clinical age groups Indian SLPs may encounter, there exist limited reported studies on SLPs managing adolescents with language impairments. Hence, the current study aimed to survey the perspectives of SLPs working in India towards the assessment of adolescents with language impairments. Therefore, the objectives of the study included (1) developing a questionnaire to target the perspectives of Indian SLPs towards the assessment of adolescent language; and (2) exploring the perspectives of the SLPs on the same.

## METHODS

The current study was a self-reported internet-based study following a cross-sectional design and a convenience sampling method. The study was done by the Helsinki Declaration of 1975, as revised in 2008. It was approved by the Institutional Ethical Committee (IECKMCMLR-09/2020/265) of Kasturba Medical College, Mangalore, Manipal Academy of Higher Education.

### Participants

The developed survey was sent out to 158 Indian SLPs, out of which a total of 102 responded. The mean (SD) age of the participants was 23.6 (2.6) within the range of 20 and 40 years of age. Table 1 depicts the demographic details of the participants.

**Table 1:** Demographic details of participants of the current study

Demographic Variables	Characteristics	Frequency (n)	Percentage (%)
Gender	Male	11	10.7%
	Female	91	89.2%
Qualification	BASLP	74	73.5%
	MSc. SLP	26	25.4%
	PhD	2	1.9%
Clinical experience	Fresh professionals	61	59.8%
	Experienced professionals	33	32.3%
	On a break	2	1.9%
	Others	6	5.8%
Work setting	University	63	61.7%
	Clinic	12	11.7%
	Hospital	7	6.8%
	Institute	7	6.8%
	Rehabilitation Centre	3	2.9%
	School	2	1.9%

**Note:** Fresh professionals included graduate students of Bachelors in Audiology and Speech Language Pathology (BASLP) and who are currently pursuing post-graduation in Speech Language Pathology (MSc. SLP)

The inclusion criteria included SLPs with a minimum of 1 year of work experience in either clinical/ institute/ hospital/ school/ university/ rehabilitation center or any other similar setups. Additionally, SLPs who had a degree in Bachelor of Audiology and Speech Language, or individuals pursuing a Master of Science in Speech Language Pathology were included in the study. The exclusion criteria included students pursuing Bachelor of Audiology and Speech Language Pathology, and SLPs practicing outside India.

### Procedure

The present study was conducted in 3 phases. Phase 1 comprised developing the questionnaire that aimed to tap upon the perspectives of SLPs towards the assessment of adolescent language; phase 2 included the data collection; and phase 3 comprised data and statistical analysis.

Phase 1: Development of the Questionnaire

The questionnaire was initially constructed with 22 items after reviewing the existing literature on adolescent language. Relevant journal articles and books on language development were primarily used for the initial construction of the questionnaire. The items in the questionnaire targeted sensitive questions that may help invoke the knowledge, practices, and attitudes of Indian SLPs towards the assessment of language in adolescents. This initial developed questionnaire was subjected to content validation by 5 experienced SLPs in the field of language development. The expert's content validated the questionnaire using a 4-point rating system (1-relevant, 2-somewhat relevant, 3-quite relevant, and 4-relevant). An average of the obtained scores (3.9) was obtained (with a content validity index of 1), indicating an excellent content validity of the developed questionnaire. After incorporating the suggestions and comments of the experts, the final version of the questionnaire included 9 questions (8 open-ended and 1 closed-ended) which were then converted into a Google form for data collection. Each of the questions was prepared to suit Indian SLPs considering the work settings, service delivery methods, assessment tools used by them, and the challenges they face while handling adolescents with language impairments. The final questionnaire is shown in the Appendix A.

Phase 2: Data Collection

The survey was sent to SLPs in India via WhatsApp, Facebook, LinkedIn, and e-mail. A web link to the survey was sent to all the participants, which comprised of an introductory message inviting the respondents to participate in the survey, followed by informed consent to participate in the study, along with instructions to complete the survey. In addition, the details of the survey and the link to the survey were posted on the research request webpage of the Indian Speech and Hearing Association, and reminders were sent every week for 2 months.

Phase 3: Data and Statistical Analysis

The retrieved data was qualitatively and quantitatively analyzed. Responses for the open-ended questions underwent qualitative (response categorization) and quantitative (descriptive statistics) analysis. A similar quantitative analysis was followed for the responses for the closed-ended questions. The descriptive statistics included determining the mean and SD for continuous variables, and frequency and percentage for discrete variables.

## RESULTS

The results of the study have been discussed as follows:

### Adolescent age range and later language development

A total of 73.5% of the participants indicated the period of adolescence to begin between 10 and 13 years of age, while 11.7% indicated it to start between 14 and 16 years of age. Alternatively, 7.8% indicated the inception to be between 17 and 19 years of age, 4.9% indicated it to occur after 20 years of age, while 1.9% stated it to commence before 10 years of age. Furthermore, 77.4% of the participants indicated the termination of the adolescent period to happen between 17 and 20 years of age. However, 11.7% indicated it to be between 13 and 16 years of age, 2.9% claimed it to end between 21 and 24 years of age, while 6.8% directed it to conclude only after 24 years of age. Table 2 illustrates the total number (percentage) of SLPs who perceived the period of termination of language development in one’s lifetime.

**Table 2:** Perspectives of Speech Language Pathologists towards the age of termination of language development

Period of termination	N (%)
Childhood	25 (24.5)
Adolescence	36 (35.29)
Adulthood	12 (11.76)
Older adulthood	2 (1.96)
Never ends	24 (23.52)
Not sure	3 (2.94)

### Targeted domains of language assessment in adolescents

**Figure 1:**
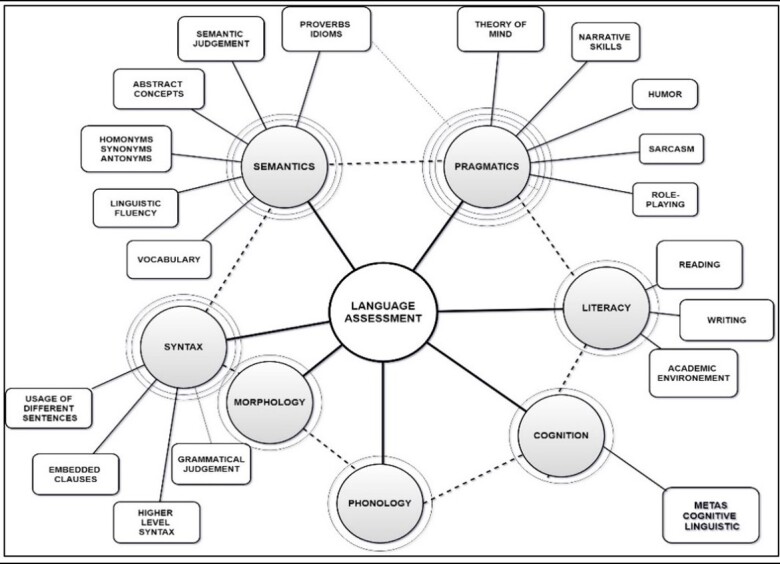
The perspectives of Speech Language Pathologists on the areas crucial for a language assessment in adolescents

**Note:** The grey shaded circles indicate the six major domains of assessment that was perceived by the participants. The number of concentric circles around each of the grey circles denotes the priority level (i.e., the more the concentric circles present, the higher priority given) indicated by the participants. The rectangular boxes indicate the sub-domains under the respective major domain that was perceived by the participants. The dotted lines do show the relationship between the major domains

Figure 1 illustrates the essential components of an adolescent language assessment from the perspectives of SLPs represented in a concept map.

The highest number of responses (79.4%) were received for considering pragmatics an important component in adolescent language assessment. This was followed by syntax (75.4%), semantics (66.6%), morphology (27.4%), phonology (20.5%), metas (15.6%), cognition (10.7%), and literacy (7.8%). A total of 5.8% of the participants reported an overall language assessment (inclusive of all domains) as crucial, while the rest 6.8% indicated other areas of assessment such as speech and psychological aspects to be vital for assessment.

Roughly 92% of the participants reported using formal testing methods such as the Linguistic Profile Test and the Manipal Manual of Adolescent Language Assessment; while 19.6% used informal methods such as narration and conversational-based tasks. A total of 34.31% of the participants reported the use of tools such as the Western Aphasia Battery and the Frenchay Dysarthria Assessment (which is ideally not considered adolescent language tests). A small proportion (3.92%) of participants reported being unaware of the relevant language assessment tools/informal methods used during the assessment of adolescents with language impairments.

### Language impairments in adolescents and their challenges

A total of 49 (48.03%) SLPs perceived language impairments to stand alone without a causative medical diagnosis, while 26 (25.49%) did not agree. However, 27 (26.47%) of the participants were unsure of the same.

#### Speaking difficulties encountered in adolescents with language impairments

A majority of the responses revealed adolescents with language impairments face difficulties predominantly in the area of pragmatics (47%) (i.e., difficulties following conversational rules), the overall language structure (35.2%), semantics (16.6%) (i.e., difficulties in the usage of vocabulary), phonology (3.9%) (i.e., exhibiting phonological errors), literacy (8.8%) (i.e., difficulties in reading and writing), metas (6.8%) (i.e., difficulties to ponder upon the content being delivered), and cognition (5.7%). Other responses did include emotional disturbances and shyness (21.5%) and speech-related issues (9.8%), while a negligible number (0.9%) reported no major effect on speaking abilities.

#### Reading difficulties encountered in adolescents with language impairments

Major deficit areas were reported under reading comprehension (51.9%), followed by difficulties in phonology (31.3%) (i.e., difficulties in decoding the sound structure of the word), morphology (11.7%) (i.e., difficulties in decoding the word structure and its meaning), semantics (8.8%) (i.e., difficulties in the usage of appropriate vocabulary), syntax (11.7%) (i.e., difficulties in following grammatical rules), and pragmatics (4.9%) (i.e., difficulties in following the context of the text being read). A total of 10.7% reported rate and fluency-related difficulties, with a few other responses indicating difficulties with cognition (4.9%) and metas (1.9%) (i.e., difficulties in the ability to reflect or manipulate the content read).

#### Writing difficulties encountered in adolescents with language impairments

The major responses reported to include deficits/errors in syntax (35.2%) (i.e., difficulties in adhering to grammatical rules), spelling/morphological (34.3%) related errors, phonology (25.4%) (i.e., difficulties in encoding of words), semantics (11.7%) (i.e., having poor lexical density and diversity), legibility (10.7%), and cognition (9.8%). A less number of deficits were reported to be related to comprehension (5.8%), overall language (4.9%), and metas (1.9%) (i.e., difficulties to reflect or think about what is written). Another major deficit area reported was the writing speed and following of commands (26.4%). Two percent of the participants reported having no or minimal writing-related deficits (1.9%), while few were unsure of the same (3.9%).

#### Other challenges faced during adolescent language assessment

**Figure 2:**
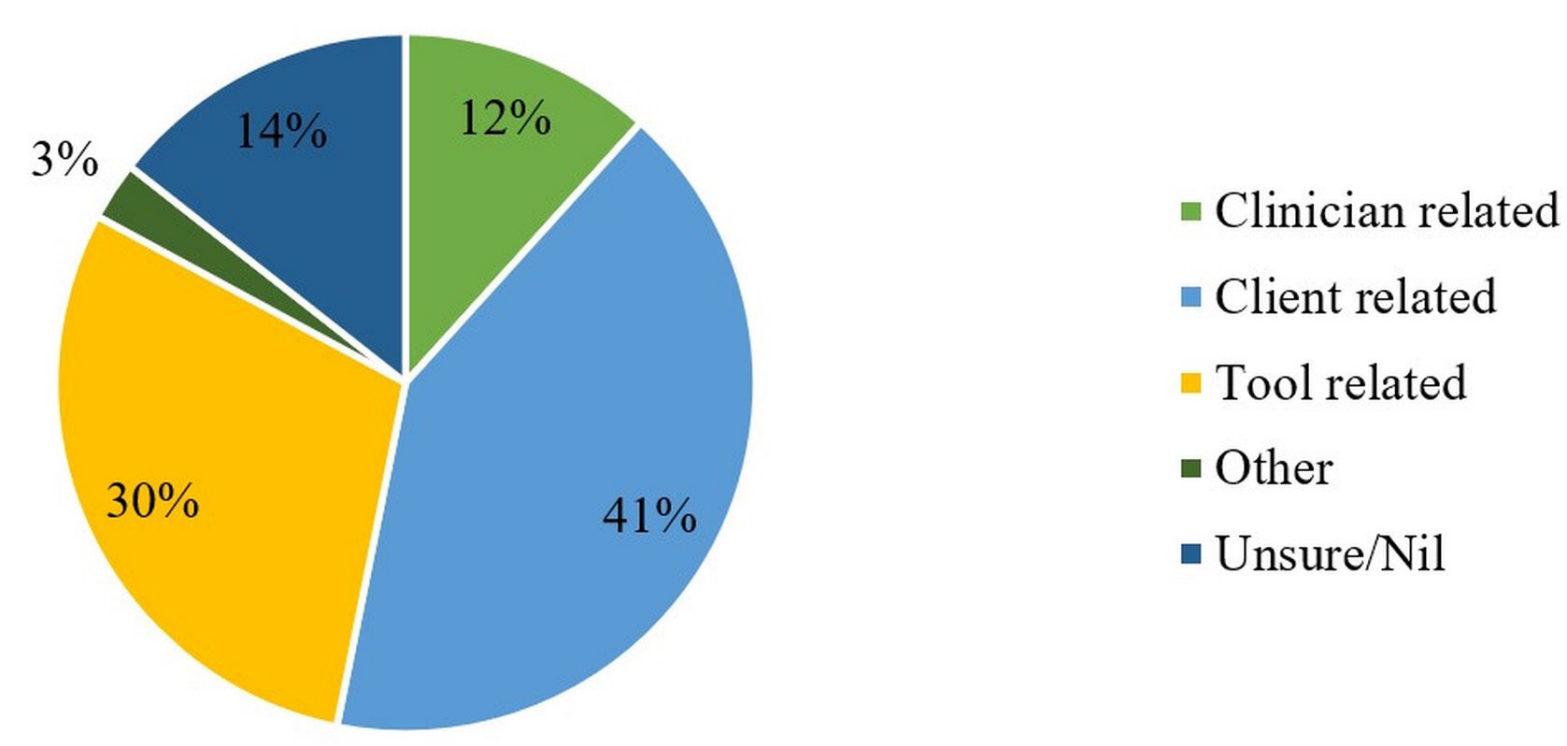
The perspectives of Speech Language Pathologists towards the challenges faced during an adolescent language assessment

Figure 2 illustrates the total percentage of SLPs who addressed the difficulties faced by clinicians during the assessment of adolescents with language impairments

The major challenges faced by the participants were client-based difficulties such as shyness, lack of cooperation, and language barriers. The tool-based challenges included the lack of standardized Indian tools, unavailability of existing western and local standardized tools. Clinician-related challenges included being unaware of the existing tools, having poor motivation in assessing adolescent clinical groups, and having language barriers. Other challenges reported included clinicians to have a poor theoretical background and clinical exposure to adolescent language development and assessment.

## DISCUSSION

The results of the study are discussed as follows.

### Adolescent age range and later language development

The responses obtained about the age range of adolescents varied from a period that begins well before 10 years of age and extending up to 50 years of age. In the current study, 59.8% of the participants were fresh professionals having less than a year of clinical experience; while around 32.3% were professionals with >1 and <10 years of experience. These less experienced fresh professionals forming the major bulk of the sample contributed to the major lack of consensus observed towards the adolescence period. Such variations in the responses that have been attributed to the experience of the respondent have been previously reported by Ebert and Kohnert^(15)^. They indicated that the key aspect of being an effective clinician is only through experience. Moreover, the period of adolescence has been used differently in various contexts and situations, mostly to suit one’s needs. Both the World Health Organisation and the Ministry of Health and Family Welfare, and countries such as Bangladesh and Africa consider the adolescence period to be between 10 and 19 years of age. Contrastively, New Zealand uses terms such as ‘youth’ and ‘adolescence’ synonymously, referring to ‘young people’ ranging between 10 and 24 years of age. Various researchers have studied adolescents who were considered to be aged between 10 and 24 years^(16)^, 13 and 19 years^(17)^, and 10 and 16 years^(7,9,18)^. Such a blend of use of adolescent periods does imply that it is conveniently decided and followed upon, with the precise onset and offset being difficult to be determined^(1)^, with no uniform pattern being recorded.

An overall level of uncertainty and poor consensus prevailed among the SLPs on the termination of language development. The SLPs in the current study indicated the development of language to terminate either during childhood, adulthood, or later adulthood. Findings of studies on language development reveal language development to be an ongoing process that may actually never end^(7-9,18)^, which was also reported by a small proportion (24%) of participants in the current study. Contrastively, other research states language acquisition to begin during infancy, peaking during childhood, where there is maximum development of language due to supporting biological factors, and ending well before adolescence^(19)^. The less experienced fresh professionals (59.8%) in the current study were unable to ascertain the termination of language development, possibly attributing it to their lack of clinical experience and confidence. This was corroborated by Ebert and Kohnert^(15)^ who felt experience to be the key aspect of being an effective clinician.

### Targeted domains of language assessment in adolescents

The distribution of responses illustrated in Figure 1, illustrates the varying perspectives of SLPs towards the crucial areas of language assessment in adolescents. As observed, all obtained responses revolved around the seven major domains (pragmatics, semantics, syntax, morphology, phonology, cognition, and literacy). Pragmatics was the most reported component (79.4%) deemed to be crucial for a language assessment in adolescents, which included areas of assessment such as humor, sarcasm, role-playing, theory of mind, and narrative skills. On a similar note, Joffe and Black^(20)^ reported adolescents to exhibit better social, emotional, and behavioral skills, with these skills being recommended to be included as part of a routine pragmatic assessment. The participants in the current study also considered the use of proverbs and idioms to be crucial for assessment. Although these figurative expressions have been considered to be an integral part of pragmatics, some do consider them to be a giant lexical unit (semantics)^(21)^. The unanimity among the responses towards pragmatics being a crucial part of assessment could be due to its explicit manner language manifestations, i.e., based on the patterns of social interaction, and preferences and interests of the individual^(22)^.

The assessment of morpho-syntax was deemed to be the second most crucial aspect of language assessment in adolescents, with areas including grammaticality judgment and use of higher-level grammar (i.e., embedded clauses and different sentences) being reported by the participants of the current study. Apart from studies that have used morphological derivations as a potential marker^(10)^, others have used measures such as T-units and C-units to assess the syntactic development in adolescents^(7)^. The semantic domain was reported to be the third most crucial aspect of assessment which included the use of linguistic fluency, complex vocabulary, semantic knowledge (breadth and depth), abstract concepts, expression of homonyms, synonyms, antonyms, proverbs, and idioms (sometimes also considered as a pragmatic skill). Similar use of such methods has been reported in other studies^(4,8)^. Vocabulary, which is considered a cornerstone, upon which other linguistic units are built, appears to grow across one’s lifespan, resulting in the usage of homonyms, antonyms, and synonyms, indirectly helping in the comprehension of metaphors and other complex linguistic units. Another aspect crucial to language assessment reported by the participants included the component of literacy (reading, writing, and academic environment). Studies have assessed language using written narratives, reading, and curriculum-based tasks^(8,11)^. The least reported areas of assessment by the participants of the current study were aspects of cognition and phonology. It was observed that the obtained responses from the present study were not influenced by the clinical work setting of the professional, but rather the experience of the professional, with the more experienced professionals providing elaborate responses than the fresh professionals.

The participants indicated the use of only two major assessment tools for adolescent language, which were the Linguistic Profile Test and the Manipal Manual of Adolescent Language Assessment. Currently, no other tools happen to be clinically and culturally appropriate to be used in India, apart from these two. Although certain SLPs had indicated the use of specific western assessment tools such as the Clinical Evaluation of Language Fundamentals and the Test of Pragmatic Language, the credibility of its use is highly questionable due to the linguistic and cultural disparity. Additionally, a large proportion of SLPs indicated the use of tools such as the Western Aphasia Battery and the Frenchay Dysarthria Assessment, which happen to be tools to assess adult communication caused due to acquired neurological conditions. Reporting non-adolescent assessment tools do imply the lack of awareness among SLPs towards this aspect. With the unavailability of standardized assessment tools, SLPs largely settle for the use of informal methods of evaluation, which was reported by the participants in the current study. A similar use of such informal methods (as mentioned in the results) for evaluation has been reported by other studies^(8,9)^. Although there have been explicit views on the use of formal and informal methods of evaluation, using a combination of both has shown promising results^(4,8,9,22)^.

### Language impairments in adolescents and their challenges

Although a large proportion (48%) of the participants agreed on language impairments to exist without a primary medical diagnosis, the rest have reported otherwise. Since the majority of language impairments have a behavioral origin, there does not exist a label that may validate this^(23)^, thereby implying language impairments to occur without a primary medical condition. Such adolescents with impairments are considered to possess an invisible disability, with these individuals passing off as poor performers, underachievers, or shy teens^(24)^. Often adolescents with language impairments manifest as late talkers, slow learners, and individuals with behavioral deviancies ending up as juvenile delinquents. These adolescents are sometimes termed as uninterested, mischievous, and troublemakers, with such misleading labels masking the impairment and amplifying the behavior associated with it. Only a detailed cognitive-linguistic evaluation done by an SLP that includes auditory-visual communication through verbal and non-verbal modes, intentionally assessing all components of language and literacy can expose this invisible disability in the adolescents.

### Speaking difficulties encountered in adolescents with language impairments

With pragmatics being a major area of difficulty reported, the adolescents with language impairments encountered difficulties in communicating their needs, conversing, quick thinking, verbal reasoning, deciphering emotions and conversation cues from the communication partner, and having circumlocutory behaviors. Pragmatic skills emphasize their importance in communication and its overall effectiveness in using other language components. Such a dominant representation of pragmatics as an affected spoken language component can be attributed to its overrepresentation when considering the perceivable developments in the aspects of humor, sarcasm, use of slang words, etc. This dominance of the pragmatic component in speaking does tend to overshadow the display of other domains (overall language, cognition, and metas), with the latter having a direct or an indirect influence on pragmatics^(25)^. Knowing that school-based SLPs are more exposed to adolescents with language impairments^(13)^ than the SLPs who tend to work in other settings, the spoken language manifestations reported by the SLPs becomes underrepresented as the total number of school-based SLPs in the current study were only 1.9%. Additionally, receiving an adolescent with a language impairment through a well-informed and intentionally designed referral system is crucial^(14)^, which may not have been observed in the current study.

### Reading difficulties encountered in adolescents with language impairments

As reported in the current study, the SLPs perceived adolescents with language impairments to exhibit deficits in the decoding of phonological rules during reading, which is in line with the findings of Tambyraja et al.^(26)^ who stated that successful reading relies on one’s code-based skills such as phonological awareness, letter-sound correspondence, alphabet awareness, and print concept. While a small proportion of participants confessed of being unaware of the type of reading deficits encountered in adolescents with language impairments, others reported the existence of psychological issues such as anxiety, shyness, and frustration associated with reading. On similar lines, Undheim and Sund^(27)^ indicated that these adolescents exhibited anxiety, depression and lack of attachment, when compared to adolescents who read effortlessly. The lack of consensus by the participants on the type of reading difficulties faced by adolescents can be attributed to the overall lack of awareness of reading-related issues associated with language^(28)^, that takes precedence during adolescence.

### Writing difficulties encountered in adolescents with language impairments

Participants indicated the presence of a large range of writing-related deficits, as indicated in the results. Similar findings have been reported by Kippin et al.^(11)^ who found adolescents with language impairments to possess poor cognitive and motor demands for spelling, handwriting deviancies, poor understanding of sentence boundaries, weak content structures, poor use of complex sentences, reduced lexical diversity, reduced ability to infer meaning, overall poor narrative skills, lack of macrostructure or plan in the content, and a lack of cohesiveness in written text. Around 12% of the participants indicated the existence of poor lexical density and diversity in written language, stating that the expansion of literate vocabulary during adolescence is well reflected in written language. Others reported writing-related issues were the manifestation of errors of phonologic, semantic, and syntactic, along with the presence of cognitive (metalinguistic and metacognitive)^(11)^, and comprehension-based issues. Although the current study observes an over-representation of spelling errors, which happens to be a minor aspect of written language deficits, this could be attributed to the lack of awareness of the other aspects of written language due to the participation of a high number of fresh professionals in the current study.

### Other challenges faced during adolescent language assessment

The obtained responses indicate that the type of difficulties faced by SLPs are clustered around client and tool-related factors^(26)^, while others were clinician-related^(28)^. Tool-related factors included the lack of awareness of existing tools^(28)^, and the lack of availability of linguistically and culturally standardized adolescent language assessment tools (both western and Indian), both of which being under-addressed dilemmas. Although attempts are underway to study the assessment of language in Indian adolescents^(7-9,18)^ using formal and informal methods, the participants report such tools being too complex and time-consuming. Apart from this, certain client-related factors included the patient’s reluctance to talk, shyness, improper furnishing of details by the family/self, and the patient’s inability to comprehend instructions. Ehren^(13)^ emphasized the importance of being aware of our professional calling and duties, and, educating the family and the teachers’ role in adolescent language development. The lack of exposure to adolescents with language impairments, unavailability of translators during the assessment process^(29)^, lack of confidence and experience in the area, and the difficulty in diagnosis^(28)^, were the clinician/self-related factors. Although a certain proportion of SLPs reported no major client-related difficulties, some mentioned difficulties in comprehending the client’s speech, and their requirement to have instructions repeated.

With the current study having used a relatively smaller sample size majorly comprising of immediate undergraduate students, future studies could overcome this issue using a larger proportion of professionals working in the field. The findings of the survey could also have probably steered the study towards an alternative course, preserving a good balance of experienced and fresh professionals. Prospective research could plan to target the perspectives of SLPs on the management strategies used to address the adolescent population.

## CONCLUSION

The results of the current study do reiterate the viewpoint of adolescents being an underserved population, thereby indicating a major lacuna among Indian SLPs towards the clinical practices for this population. The study concludes by suggesting the importance of conduction of intensive awareness programs for Indian SLPs regarding adolescent language development and assessment.

## Appendix A Questionnaire to invoke the knowledge, practices, and attitudes of Indian SLPs towards the assessment of language in adolescents

**Table:**

**Sl. no.**	**Questions**
Q1	What age range do you feel adolescents would fall under?
Q2	At what age do you feel that the language development would end?
Q3	What are the areas of language assessment that you feel are crucial in adolescents?
Q4	Do you feel that language impairments observed in adolescents can stand independently without a causative medical diagnosis?
Q5	Which are the adolescent language assessment tools/informal methods that you use to assess adolescents with language impairments?
Q6	What are the difficulties you feel that adolescents with language impairments may face while speaking?
Q7	What are the difficulties you feel that adolescents with language impairments may face while reading?
Q8	What are the difficulties you feel that adolescents with language impairments may face while writing?
Q9	What are the difficulties you have faced during the assessment of adolescents with language impairments?
